# Author’s Reply—A double-blind case study?

**DOI:** 10.1016/j.hrcr.2021.02.001

**Published:** 2021-02-06

**Authors:** Xiaoke Liu, Shiau-Ing Wu

**Affiliations:** ∗ Division of Cardiology, Ascension Borgess Hospital, Kalamazoo, Michigan, and Department of Medicine, Western Michigan University, Homer Stryker M.D. School of Medicine; † United Health Services Hospitals, Wilson Medical Center, Johnson City, New York

We read with great interest the letter to the editors entitled “A double-blind case study?”, authored by Drs Leung and Gallagher. It is unclear to us why the authors called the case “double-blind” when the position of the esophagus was clearly visualized by the CARTO 3-D mapping system as well as the intracardiac ultrasound. In other words, prior to esophagus deviation, we can “see” the esophagus right under the ablation catheter tip, while the esophagus is nowhere to be found in the immediate vicinity of the catheter tip on the intracardiac ultrasound post deviation. It seemed straightforward to us and to the 2 experts that peer-reviewed the paper. We did not empirically do endoscopy just to “see” the esophagus for EsoSure-related injury because we believe instrumentation using the rigid endoscopy probe right after ablation may cause further injury to the esophagus and may not be ethical.

The reason we chose to use the EsoSure device was based on the DEFLECT GUT study, which enrolled 687 patients, and the ease of use that fits our workflow.^1^ The DEFLECT study did not show any serious device-related complication but demonstrated much lower esophagus temperature rise compared to the control arm. Given the extremely low incidence of atrial esophageal fistula (∼0.1%), it is likely impossible for any intervention to show a significant difference in its incidence in a clinical trial, let alone comparing the superiority of one method over the other. The individual trials the authors cited looked at esophageal thermal injury, which is not the same as atrial esophageal fistula, as the vast majority of the thermal injuries identified on endoscopy do not evolve into atrial esophageal fistula and should be interpreted with this in mind. Despite anecdotal reports of EsoSure-related mechanical injury to the esophagus, we are unaware of any mortality directly attributed to the use of EsoSure, bearing in mind the complication this device was designed to prevent is frequently fatal.

We have no financial association with any companies that manufacture esophagus protection devices, unlike Drs Leung and Gallagher, and we do not advocate one method over another for reasons discussed above. The readers should choose a particular type of method for esophagus protection that is based on clinical studies and the one that suits their ablation styles.
